# Intrinsically ionic conductive cellulose nanopapers applied as all solid dielectrics for low voltage organic transistors

**DOI:** 10.1038/s41467-018-05155-y

**Published:** 2018-07-16

**Authors:** Shilei Dai, Yingli Chu, Dapeng Liu, Fei Cao, Xiaohan Wu, Jiachen Zhou, Bilei Zhou, Yantao Chen, Jia Huang

**Affiliations:** 1 Interdisciplinary Materials Research Center, School of Materials Science and Engineering, Tongji University, 201804 Shanghai, China; 2 Key Laboratory of Inorganic Functional Materials and Devices, Shanghai Institute of Ceramics, Chinese Academy of Science, 201804 Shanghai, China

## Abstract

Biodegradability, low-voltage operation, and flexibility are important trends for the future organic electronics. High-capacitance dielectrics are essential for low-voltage organic field-effect transistors. Here we report the application of environmental-friendly cellulose nanopapers as high-capacitance dielectrics with intrinsic ionic conductivity. Different with the previously reported liquid/electrolyte-gated dielectrics, cellulose nanopapers can be applied as all-solid dielectrics without any liquid or gel. Organic field-effect transistors fabricated with cellulose nanopaper dielectrics exhibit good transistor performances under operation voltage below 2 V, and no discernible drain current change is observed when the device is under bending with radius down to 1 mm. Interesting properties of the cellulose nanopapers, such as ionic conductivity, ultra-smooth surface (~0.59 nm), high transparency (above 80%) and flexibility make them excellent candidates as high-capacitance dielectrics for flexible, transparent and low-voltage electronics.

## Abstract (web summary)

Next-generation organic electronics require flexible organic field effect transistors that show low-voltage operation and are biodegradable. Here, Huang and co-workers demonstrate high-performance transistors that utilize solid-state ionic conductive cellulose nanopaper as the dielectric.

## Introduction

Rapid technological progress has led to a significant reduction in the life expectancy of consumer electronic products, accelerated the consumption of non-renewable resources, and generated electronic-waste (e-waste) at an alarming rate^1^. E-waste can lead to adverse human health effects and severe environmental pollution^2^. Therefore, development of green electronic devices is of great significance because green electronics represents not only a novel scientific term but also an emerging area of research aimed at identifying compounds of natural origin that have applicability in environmentally safe (biodegradable) and/or biocompatible devices^3–5^. Moreover, low-voltage operation of electronic components is important to obtain energy-efficient electronics^6^. Organic field-effect transistors (OFETs), as promising components in future flexible, wearable and portable electronic devices, have numerous potential applications in sensors^7–10^, radio-frequency identification tags^11,12^ and flexible displays^13^. Till date, most flexible OFETs have been prepared using polymer acting as insulators and substrates without considering the environmental problem^14–16^. However, poor biodegradability of these polymers may ultimately lead to environmental problems. Moreover, most of the polymeric insulators have low dielectric constant which may not suitable for the fabrication of low-voltage OFETs. Therefore, the development of green, environment-friendly and flexible substrate and dielectric materials for the fabrication of low-voltage OFETs is of great significance and highly desirable.

Papers as ubiquitously used materials in everyday life have attracted intensive interests because they are low-cost, renewable, biodegradable and flexible^17–19^. Some promising electronic applications based on paper have been reported in literature studies^17,20–28^. However, the porous structure and large surface roughness of ordinary paper make it challenging to fabricate electronic devices directly on its surface. Therefore, an additional coating layer is required for smoothing the surface of paper^24,28^. Although E. Fortunato et al. have done excellent works in using cellulose paper or ordinary paper to fabricate oxide semiconductor field-effect transistors, the application prospects of these papers are still limited by their large surface roughness^23,25^. For example, their papers cannot be used directly as dielectric materials for OFETs because organic semiconductors are very sensitive to surface defects. Therefore, paper with low surface roughness are still highly needed for organic electronics. Recently, nanopapers, including cellulose nanopapers, carbon nanopapers, inorganic nanopapers, and electrospun nanopapers, have been successfully prepared for flexible electronics^29^. Among them, cellulose nanopaper, fabricated using nanofibrillated cellulose (NFC), can be used directly as a substrate material due to its low surface roughness^30–32^. We have reported transparent and flexible transistors using cellulose nanopaper acting as substrate and achieved good device performances^30^. Compared with ordinary paper, the lower surface roughness of cellulose nanopapers promises it a broader application prospect in the field of electronics, including but not limited to oxide semiconductor based electronics.

In practical applications, reduction in the operating voltage of electronic components is an important approach to obtain low power consumption electronics^6^. Conventionally, low-voltage OFETs can be achieved by reducing the thickness of the dielectric layers or using high dielectric constant (high-k) dielectric materials^33,34^. Ultra-thin polymer layers and self-assembled mono-/multi-(SAM/SAMT) layers have been used as ultra-thin dielectric layers^33,35–37^. Nevertheless, most polymer dielectrics are not degradable, and reduction in the thickness of polymer insulator layers may induce problems associated with gate current leakage, in particular, for large area devices. The formation of SAM/SAMT layers usually relies on specific surface chemistry, therefore the choice of substrates for this approach is limited, especially for flexible electronics. High-k dielectric oxide materials, such as zirconium oxide (ZrO_2_), hafnium oxide (HfO_2_) and sodium beta-alumina have also been demonstrated to reduce the operating voltage for OFETs^33,38,39^. These inorganic materials, in general, require a high annealing temperature and complex preparation process, which may not compatible with many electronic fabrication processes. Moreover, the rigidity of these materials limits their practical applications in flexible electronics. Recently, low-voltage FETs based on electrolyte-gate dielectrics have been reported^40–44^. However, these materials usually contain ionic liquid or gel, which makes them difficult to be employed in all solid devices, and limits their potentials in ultrathin devices and flexible electronics.

In this study, we demonstrated the intrinsic ionic conductive property of (2, 2, 6, 6-tetramethylpiperidin-1-yl) oxidanyl (TEMPO) oxidized nanocellulose and further used it to fabricate solid-state ionic conductive cellulose nanopapers (ICCNs). The intrinsic ionic conductivity of ICCNs originated from the migration of sodium ions, which were introduced during TEMPO oxidation process. Moreover, ICCN also exhibited ultra-smooth surface. We demonstrated low-voltage flexible OFETs by using the all solid-state ICCNs acting as both the gate dielectric and substrate. Different types of organic semiconductors (OSCs) including p-type small molecule 2,7-dioctyl [1] benzothieno[3,2-b] [1] benzothiophene (C8-BTBT), n-type small molecule *N*, *N*-dipentadecafluorooctyl-1,4,5,8-naphthalene tetracarboxylic diimide (NTCDI-F15) and polymer semiconductors poly (3,3′′′ -didodecyl quarter thiophene) (PQT-12) were utilized for these low-voltage OFETs. All these OFETs devices could be operated at gate-source voltage below 2 V. The low operating voltage could be attributed to the formation of an electric-double-layer (EDL)^45^ at the interface between dielectric layer and OSC due to the presence of sodium ions in the ICCN. High transmittance of ICCN and C8-BTBT composite film indicates that devices fabricated in this study also have potential applications in future transparent electronics. High flexibility of our OFETs was demonstrated by measuring these devices at different bending radii, and no discernible current change was observed when the radius of the device was bent to as small as 1 mm. In order to further evaluate the potential applications of ICCN as dielectric materials, organic complementary inverters based on ICCN were fabricated, which exhibited low operating voltages and showed good voltage transfer characteristics (VTCs). By integrating the advantages including low surface roughness, transparency, flexibility, and solid-state ionic conductivity, ICCNs thus possess broad applications in future biodegradable, flexible, transparent, and low-voltage electronics, including but not limit to organic electronics.

## Results

### Characteristics of ionic conductive cellulose nanopaper

**Fig. 1:**
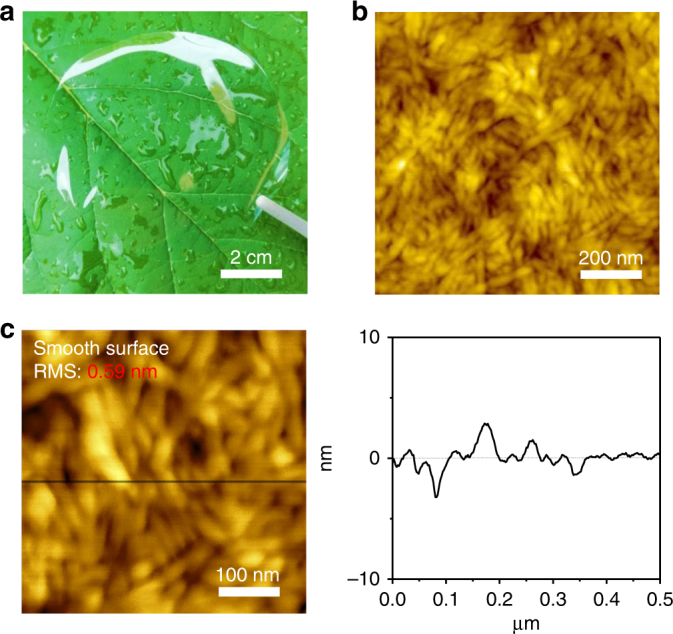
Photograph and AFM characterization of ICCN. **a** Photograph of a highly transparent 40 μm-thick ICCN. **b** AFM image of a ICCN. **c** AFM line scan of a ICCN, shows ultra-smooth surface with RMS of ~0.59 nm

ICCNs were prepared from NFC solution, treated by TEMPO oxidation process. Detailed preparation process was described in the experimental section. Figure 1a shows the transparency of ICCN, demonstrating its promising potential in all transparent electronics. Besides, ICCN has an ultra-smooth surface with a root mean square roughness (RMS) of 0.59 nm which can be observed from the line scan of the surface atomic force microscopy (AFM) image shown in Fig. 1b, c. Such a smooth surface is attributed to the compact arrangement of NFCs. The hydrogen bond and mechanical entanglement force between different NFCs are two main reasons for the compact close-packing. To further observe the morphology of the nanocellulose, AFM measurements were performed on the diluted TEMPO-oxidized nanocellulose solution (Supplementary Fig. 1). It appears that the TEMPO-oxidized nanocelluloses have diameters of 5~15 nm and lengths of 150~200 nm. Scanning electron microscope (SEM) was also carried out to study the surface morphology of ICCNs (Supplementary Fig. 2). Due to the existence of hydrogen bond and mechanical entanglement force between different NFCs, there are no obvious pores on the surface of the ICCNs. This result also confirmed that ICCNs have an ultra-smooth surface. For most of the organic electronic devices, ultra-smooth surface is essential to achieving good devices’ performance. For OFETs, the ultra-smooth surface of ICCN makes it an excellent candidate for dielectric and substrate layer.

**Fig. 2:**
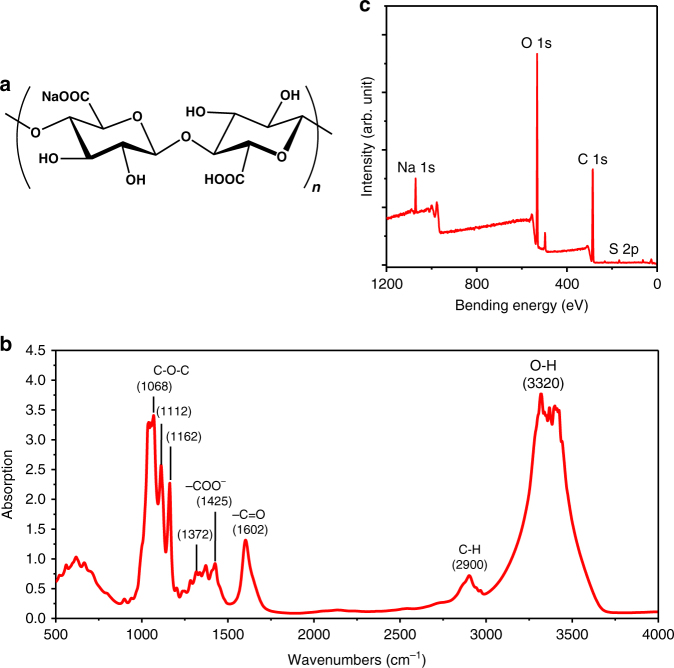
Chemical structure, FTIR and XPS analysis of ICCNs. **a** The chemical structure of ICCNs pretreated with TEMPO. The FTIR spectra (**b**) and XPS spectrum (**c**) of a 40 μm-thick ICCN

Till date, papers have been extensively studied as low-cost, biodegradable and light-weight substrates^30,46–49^. However, the intrinsic ionic conductive property of TEMPO-oxidized cellulose nanopapers has not yet been investigated. Herein, we evaluated this property and attributed it to the migration of sodium ions in cellulose nanopapers. The chemical structure of the TEMPO-oxidized NFC is shown in Fig. 2a. Fourier transform infrared (FTIR) spectroscopy measurement was carried out for ICCNs and the result is shown in Fig. 2b. The absorption spectrum shows a strong and broad band at 3100–3700 cm^−1^, which originates from stretching vibration of O–H, while weak and narrow band absorption centered at 2850 cm^−1^ is ascribed to the stretching of asymmetric and symmetric methyl and methylene C-H group^50^. Peaks at 1068 and 1162 cm^−1^ are assigned to C–O–C asymmetric stretching and bending absorption, respectively^51^. Moreover, the absorption peaks at 1500–1700 and 1250–1500 cm^−1^ are attributed to the presence of sodium carboxyl groups^52^. Energy dispersive spectroscopy (EDS) was also employed to confirm the successful introduction of sodium ions in ICCN (Supplementary Fig. 3). X-ray photoemission spectroscopy (XPS) was employed to analyze the relative concentration of sodium ions and the corresponding results are shown in Fig. 2c and Table 1. Three major peaks located at 285 eV, 532 eV, and 1071 eV are corresponding to the excitations of C (1 s), O (1 s), and Na (1 s), respectively^53^. The content of sodium ions in ICCN is about 2.97%. A possible explanation can be attributed to the partial conversion of the hydroxymethyl group at the glucose C6 position to sodium carboxylate.

**Table 1:** Element types and contents in a 40 μm-thick ICCN measured by XPS

Name	Atomic %
O 1 s	36.66
C 1 s	59.38
Na 1 s	2.97
S 2p	0.98

**Fig. 3:**
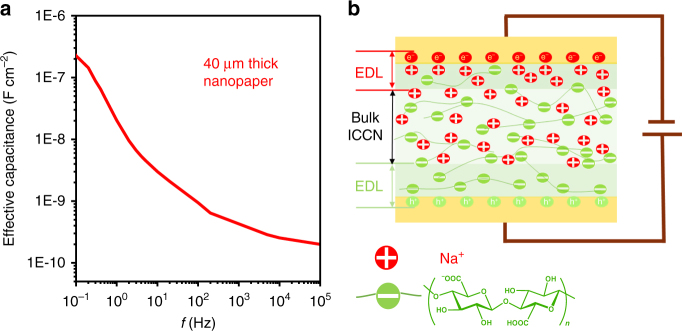
Frequency dependent effective capacitance of 40 μm thick ICCNs. **a** Frequency-dependent effective capacitance (*C*-*f*) of a 40 μm-thick ICCN measured in a metal/insulator/metal (Au/ICCN/Au) structure. **b** Schematic diagram demonstrates the formation of EDL in ICCNs under the action of electric field

High effective capacitance is one of the most important properties of dielectric materials to achieve excellent low-voltage performance of OFETs^33,39^. We examined the frequency-dependent capacitive behavior of a 40 μm-thick ICCN on metal-insulator-metal (Au/ICCN/Au) structure. The effective capacitance increases with the decrease in frequency from 100 KHz to 0.1 Hz (Fig. 3a). Corresponding effective dielectric constant is shown in Supplementary Fig. 4. The effective capacitance of this 40 μm-thick cellulose nanopaper at 0.1 Hz is 220 nF cm^−2^. This large capacitance of ICCN was attributed to the formation of EDL (Fig. 3b). The sodium ions in TEMPO oxidized celluloses could be dissociated from sodium carboxylates and migrate to the surface of ICCN under the applied electric field. Since EDL capacitance of ICCNs may not be the same when ICCNs are in contact with the semiconductors, we have examined the frequency-dependent capacitance of ICCNs in metal/ICCN/semiconductor/metal structure, as shown in Supplementary Fig. 5. The frequency-dependent capacitance behavior of ICCNs showed slightly difference when they were in contact with different semiconductors. This slightly difference may be attributed to that OSCs are permeable to ions in ICCNs. To further evaluate ICCNs as dielectrics, breakdown voltage was also measured. However, when the operating voltage reached 80 V (or −80 V), no significant damage was observed (Supplementary Fig. 6).

Thermal stability is another important property to be considered when ICCN is used as a dielectric layer. Thus, thermogravimetry (TG) analysis was carried out (Supplementary Fig. 7) to systematically evaluate this factor. A slight decrease in weight was observed below 200 °C, which might be due to the loss of moisture adsorbed in ICCN. A significant decrease in weight of ICCN starting at 200 °C indicates that the decomposition temperature of ICCN is about 200 °C. This temperature is higher than preparation temperature of general OSCs. Therefore, ICCN has excellent thermal stability which is good enough for most OFETs fabrication.

In practical applications, it is also necessary to consider the mechanical properties of dielectric materials. The mechanical properties of ICCNs were tested by using stress–strain testing apparatus. The stress–strain curve of ICCNs is displayed in Supplementary Fig. 8. The tensile strength and Young’s modulus of ICCNs are as high as 98.9 MPa and 120.7 GPa, respectively. Therefore, ICCNs possess excellent mechanical properties.

In conclusion, ICCN with high transparency, ultra-smooth surface, high dielectric constant, temperature resistance, and excellent mechanical properties can be effectively used as an excellent dielectric material for low-voltage OFETs devices.

### Low voltage organic field effect transistors

**Fig. 4:**
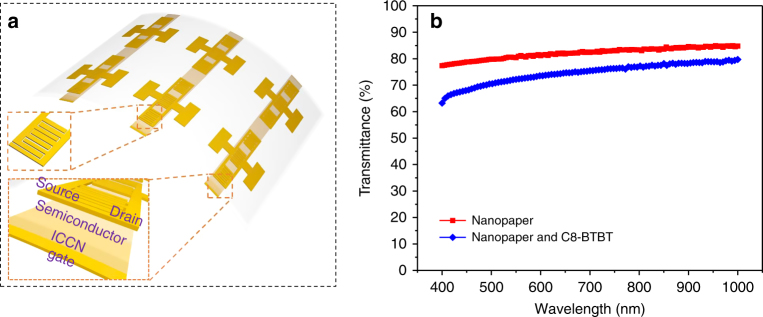
Schematic of transparent OFET based on ICCN. **a** Schematic illustration of the ICCN based OFETs with a bottom gate top contact architecture. **b** Optical transmittance of a 40 μm-thick ICCN and an ICCN/C8-BTBT composite film at different wavelengths

The 40 μm-thick ICCNs have good self-supporting properties and maintain good flexibility and transparency, therefore, they were investigated as both the dielectric layers and substrates simultaneously for the fabrication of low-voltage OFETs. Figure 4a shows the schematic illustration of ICCN-based OFETs with bottom gate, top contact architecture. Figure 4b compares the optical transmittance of both 40 μm-thick ICCN and 40 μm-thick ICCN/C8-BTBT composite film (C8-BTBT was deposited on the surface of ICCN by thermal evaporation). The transmittance of 40 μm-thick ICCN/C8-BTBT composite film showed a slight decrease compared to that of 40 μm-thick ICCN. However, both films exhibited decent transparency.

**Fig. 5:**
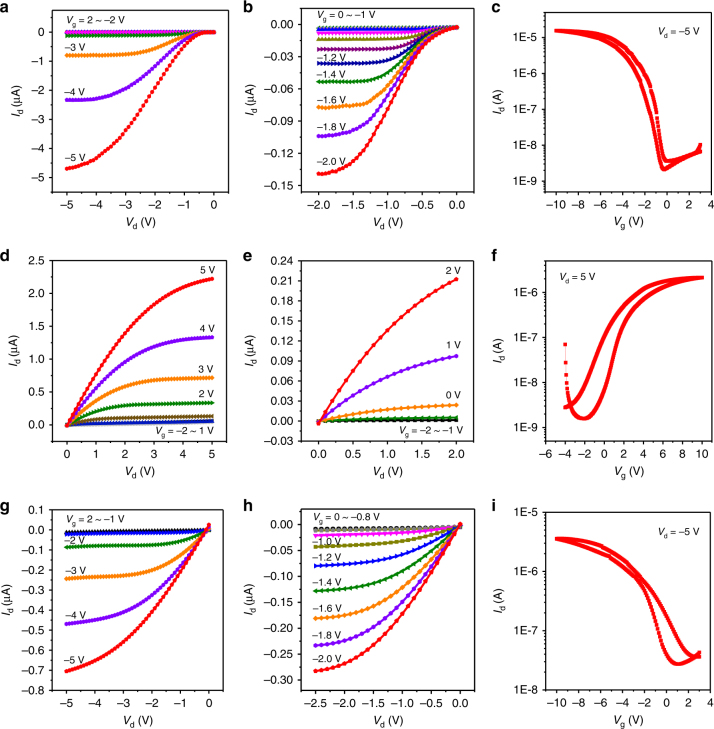
Electrical characteristics of ICCNs based OFETs. **a**, **b** *I*_d_–*V*_d_ curves and **c** transfer characteristics curves (*I*_d_*–V*_g_) of a p-channel C8-BTBT OFET. **d**, **e** *I*_d_*−V*_d_ curves and **f** transfer characteristics curves (*I*_d_*−V*_g_) of a n-channel NTCDI-F15 OFET. **g**, **h** *I*_d_*−V*_d_ curves and **i** transfer characteristics curves (*I*_d_*−V*_g_) of a PQT-12 OFET

Figure 5a shows the output *I*–*V* characteristics of a p-type C8-BTBT OFET device with operating voltage below 5 V. Both a linear and saturation regime can be observed clearly, which indicates good OFET characteristics. Figure 5b shows the output characteristics of the same device operated at voltage below −2 V. This device still maintained an excellent gate modulation property at such low voltages. The formation of EDL and hence a high local electric field at the ICCN/OSC interface leads to a large effective capacitance, which effectively reduces the voltage required to operate the OFETs. Figure 5c shows the transfer characteristics of a C8-BTBT OFET. Contact resistance was observed, which could be partially attributed to the difference between the work functions of Au source/drain electrodes (−5.1 eV) and lowest unoccupied molecular orbital (LUMO) of C8-BTBT (−5.39 eV), and it may also be affected by the C8-BTBT film morphology on the surface of the ICCN^54,55^. The on/off current ratio is higher than 10^3^ and the threshold voltage (*V*_th_) is −1.26 V. The calculated effective mobility for this device is 7.24 × 10^−2^ cm^2^ V^−1^ s^−1^. The leakage current is shown in Supplementary Fig. 9.

In addition to the p-type small molecular OFETs, we also fabricated *n*-type NTCDI-F15 OFETs based on ICCNs, and measured device performances (Fig. 5d–f). This device exhibited good gate modulation and transistor characteristics even under gate voltage below 2 V (Fig. 5e). The on/off current ratio of this device was also larger than 10^3^, and the *V*_th_ was as low as 0.16 V. The calculated effective charge mobility was 9.87 × 10^−3^ cm^2^ V^−1^ s^−1^. This indicates that ICCN can not only be used to fabricate p-type OFETs, but also be suitable for the preparation of n-type devices.

To further evaluate the range of applications with ICCNs acting as gate dielectric material, polymer semiconductor PQT-12 was also used as active layer to fabricate OFETs. The output and transfer characteristics are shown in Fig. 5g–i. The on/off current ratio of this device was about 300 and *V*_th_ was 0.28 V. The effective mobility was calculated to be around 2.13 × 10^−2^ cm^2^ V^−1^ s^−1^. As expected, this polymer device also displayed excellent gate modulation and transistor characteristics even under gate voltage below 2 V (Fig. 5h). This indicates that ICCN dielectric can be effectively used with wide variety OSC molecules, with both vacuum deposited and solution-deposited semiconductor layers. All these devices were tested in vacuum and the obtained results were summarized in Table 2.

**Table 2:** Device parameters of different OFETs using 40 μm-thick ICCN acting as dielectrics

Semiconductor	Threshold voltage (*V*_T_) (V)	Mobility (cm^2^ V^−1^ s^−1^)	On/off ratio
C8-BTBT	−1.26	7.24 × 10^−2^	6.89 × 10^3^
NTCDI-F15	0.16	9.87 × 10^−3^	2.30 × 10^3^
PQT-12	0.28	2.13 × 10^−2^	2.97 × 10^2^

Since OSCs are permeable to ions, OSCs based electrolyte-gated transistors are usually considered working in a mixed operating mode (field effect and electrochemical doping)^40^. It should be noted that the electrochemical doping of the OSCs can be suppressed by introducing proper mobile ions in the corresponding polyelectrolytes^56^. In our case, ICCNs can be regarded as p-type polyelectrolytes (Na^+^ charged cellulose nanopapers). The mobile Na^+^ in ICCNs will be attracted toward the gate electrode during the p-type transistor operation, while the carboxylic acid root ions (-COO^−^) on the main chain of the cellulose will remain fixed. This suppresses electrochemical doping of the p-type semiconductors in ICCNs based OFETs. But for ICCNs-based n-type OFETs, electrochemical doping can not be completely avoid because the mobile Na^+^ in ICCNs will migrate toward OSCs under the action of electrical field. Therefore, ICCNs-based p-type and n-type OFETs may operated in the field effect mode and mixed operating mode, respectively.

**Fig. 6:**
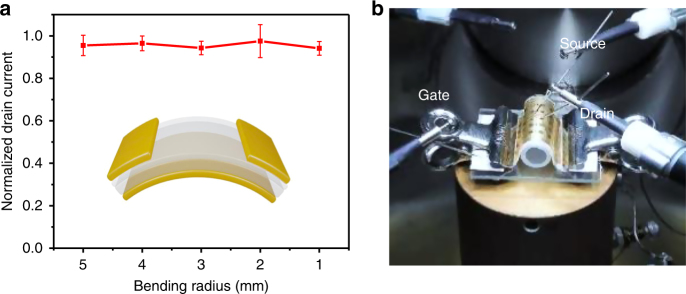
Bending tests on flexible OFETs. **a** Normalized maximum drain current at *V*_g_ of −6 V and *V*_d_ of −5 V (normalized to the initial drain current measured in the flat state) as a function of the bending radius. Error bars represent standard errors from five times independent tests of a device. **b** The picture depicts implementation of this bending test

Flexibility is one of the most important trends for future large-area electronics^3,57^. Therefore, it is essential to develop devices capable of withstanding very small bending radii without decrease in performance. Herein, we demonstrated the excellent flexibility of our devices by testing ICCNs based C8-BTBT OFETs. The bending direction of these devices was parallel to the source-drain current (strain vertical to the source-drain electrodes). In order to obtain intuitive comparison of results, the source-drain current in the bending state was normalized to that in the flat state. Figure 6 shows the normalized maximum drain current as a function of different bending radii ranging from 5 to 1 mm. Only little change was observed, even when the radius of the device was reduced down to 1 mm. To further confirm that our devices have excellent flexibility, the normalized mobility as a function of different bending radii ranging from 5 to 1 mm was also investigated (Supplementary Fig. 10). When the radius of the device reduced down to 1 mm, only a slight decrease (4.1%) in normalized mobility was observed, which indicates that our device has a wide range of opportunities for mechanical flexible electronic applications.

### Complementary organic inverter performance

**Fig. 7:**
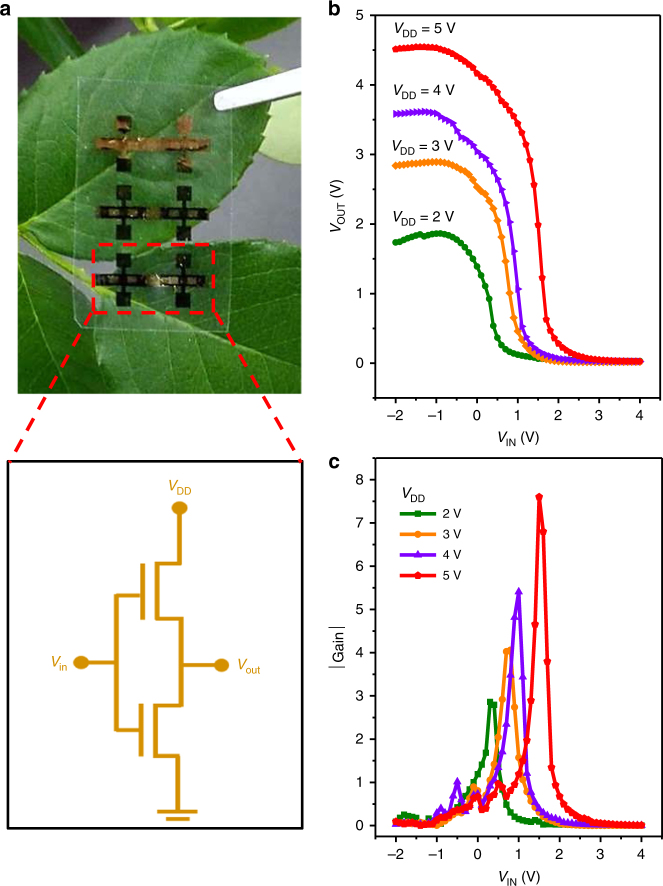
Organic complementary inverter on ICCN dielectrics. **a** Image of an organic complementary inverter and its simplified circuit diagram. **b** Output voltage and **c** gain as a function of the input voltage with supply voltage step between 2−5 V. The input voltage was swept from −2 to 4 V

The inverter, a basic unit of a logic circuit, plays extremely important role in integrated circuits. However, traditional complementary metal oxide semiconductor architecture inverters usually require a high temperature fabrication process with rigid crystalline silicon as the substrate which is not suitable for low-cost, flexible and light-weight portable electronics^58^. Implementation of organic complementary circuits based on nanopaper provides new opportunities for low-cost, light-weight and green portable electronics. Thus herein, we further fabricated organic complementary inverters with n- and p-type small molecule OFETs described above. Figure 7a shows the photo image of the inverter. Two gates of the n- and p-type OFETs were connected by conductive silver paste to form an input node while the source of the p-type OFETs was connected to the drain of n-type OFETs to provide an output node. The input voltage (*V*_IN_) was swept from −2 to 4 V and the supply voltage (*V*_DD_) was stepped between 2−5 V. The measured VTCs of the inverter along with its static gains are displayed in Fig. 7b, c, respectively. Key parameters of the inverter extracted from Fig. 7b are listed in Supplementary Table 1. Large voltage discrimination was observed between high and low states, indicating a good inverter performance. Low operation voltage (below 5 V) for the complementary organic inverter is well consistent with the low-voltage OFETs performance discussed above. A sharp transition region as low as 0.5 V was also observed. Although the gain of the complementary inverter is moderate, it can be further improved by optimizing the aspect ratio of the n- and p-type OFETs.

### Origin of ionic conductivity in cellulose nanopapers

The ionic conductivity of cellulose nanopapers plays an important role in our low voltage OFETs, it is essential to make a clear explanation for the cause of the formation of ionic conductivity in TEMPO-oxidized cellulose nanopapers. Due to the high rigidity of the main chain structure of nanocellulose, it is very difficult to transfer ions through the movement of cellulose chain. Therefore, we proposed that the ionic conductivity of the cellulose nanopaper is mainly due to the sodium ions in TEMPO-oxidized celluloses, which could be dissociated from sodium carboxylates under the help of trace amount of moisture. The hygroscopicity of the hydrogen bonds in nanocellulose chemical structure assists the presence of water in cellulose nanopapers even under a vacuum condition. Although protons might also be dissociated from carboxylate with the trace amount of moisture, the number of dissociated protons might be limited, because the carboxylic acid is a weak acid, and a lot of the carboxylates have been converted to sodium carboxylates during the preparation processes of cellulose nanopapers. In order to confirm this ionic conductive mechanism in ICCNs, we have fabricated C8-BTBT OFETs based on ICCNs washed with different times (ICCNs were prepared from nanocellulose solution with different washing times) and tested these devices in a high vacuum condition around 1 × 10^−4^ Pa. It is clear that C8-BTBT OFETs fabricated on three-time-washed ICCNs have larger drain current than C8-BTBT OFETs fabricated on six-time-washed ICCNs (Supplementary Fig. 11a). The three-time-washed ICCNs might have more sodium ions than six-time-washed ICCNs (Supplementary Fig. 11b, c). Therefore, we think that the sodium ions play an important role in the formation of ionic conductivity in ICCNs. To further explain that the dissociation of sodium ions from sodium carboxylates is under the help of trace amount of moisture, we have fabricated CYTOP encapsulated C8-BTBT OFETs with ICCNs acting as both dielectrics and substrates, to exclude the impact of air humidity fluctuations on device performances. The moisture content of the encapsulated devices is higher than that of the unencapsulated devices tested under high vacuum condition, because the trace amount of moisture present in the air will be encapsulated in ICCNs during CYTOP encapsulating process. It should be noted that the moisture content is still very low in ICCNs of the encapsulated OFETs. Supplementary Fig. 12a, 12b show the open-atmosphere output and transfer characteristics curves of a CYTOP encapsulated C8-BTBT OFET, respectively. Supplementary Fig. 12c, d show the output and transfer characteristics curves obtained from the same device in vacuum before CYTOP encapsulation, respectively. A comparative analysis of the characteristic curves before and after CYTOP encapsulation indicates that both of them exhibited decent OFETs performance. Interestingly, the device showed higher drain current after CYTOP encapsulation, as compared with the unencapsulated one. This is due to that the moisture-assisted sodium ions dissociation process enables the encapsulated ICCN to produce more migratable ions than the unencapsulated ICCN under high vacuum. Therefore, we proposed that trace amount of moisture is essential for the dissociation of sodium ions for sodium carboxylates in cellulose nanopaper, which can be further utilized to improve the performance of our OFETs.

### In-plane gate transistors

ICCNs not only can be used for the fabrication of OFETs with different types of semiconductor materials acting as channel layer, but also are suitable for the fabrication of OFETs with different device structures. We have fabricated an in-plane gate transistor based on ICCN dielectric and tested this device in air (Supplementary Fig. 13). This in-plane gate transistor can be operated below 2 V. This is mainly due to the large lateral capacitive coupling effect induced by the ionic conductivity of the ICCN. Supplementary Fig. 14 shows the vertical capacitive coupling effect and lateral capacitive coupling effect. The lateral capacitive coupling effect is very small in common dielectric materials without ions due to the large coupling distance and low dielectric constant. Therefore, it is difficult to fabricate a low-voltage in-plane gate transistor based on those dielectric materials. However, the mobile ions contained in ICCNs can migrate laterally under the transverse electric field, and hence introduce a high lateral capacitive coupling effect which is large enough to drive transistors at low voltage. The successful preparation of in-plane gate OFETs on ICCN further confirmed that ICCN has ionic conductivity.

### Cellulose nanopapers with other types of ions

For the ICCNs in this work, sodium ions contributed to the ionic conductivity of cellulose nanopapers. However, in terms of the reaction principle of TEMPO oxidation^59^, it is also possible to used other types of ions to replace sodium ions in cellulose nanopapers, such as lithium ions (Li^+^), potassium ions (K^+^). The role of TEMPO in the reaction is to convert the hydroxymethyl at the glucose C6 position into a carboxylic acid group, and the effect of sodium hypochlorite (NaClO) is to oxidize the reduced state TEMPO to its original state (Supplementary Fig. 15). The formation of sodium carboxylate in the nanocellulose is due to the use of NaClO as the oxidant for reduced state TEMPO and sodium hydroxide (NaOH) solution to adjust the hydrogen ion concentration (pH value) of the solution during the reaction. Therefore, it is possible to replace sodium ions with other types of ions during TEMPO oxidation process. In addition to TEMPO oxidation method, other chemical modification methods can also be used to introduce different ions in nanocellulose, such as strong acid hydrolysis process which will introduce protons in nanocelluloses^60^. The possibility of using other ions for the replacement of sodium ions further expands the application prospects of conductive cellulose nanopapers in the field of electronic devices.

## Discussion

In conclusion, a class of environmental-friendly and all solid-state ionic conductive cellulose nanopaper (ICCN) dielectric was demonstrated. High transparency, low surface roughness, good thermal durability, and excellent mechanical properties were observed for this dielectric material. Different types of OSCs including p-type small molecule C8-BTBT, n-type small molecule NTCDI-F15, and polymer semiconductor PQT-12 were used as active materials to fabricate flexible OFETs, with 40 μm-thick ICCNs acting as both dielectric layer and substrate layer. All these devices displayed good OFETs performance and could be operated at gate-source voltage below 2 V. This can be attributed to the ionic conductive property of ICCN which can induce an EDL and hence a high local electric field at the ICCN/OSC interface at gate voltage of just a few volts. The high flexibility of C8-BTBT OFETs with 40 μm-thick ICCN as dielectric layers was measured at different bending radii and no significant drain current change was observed compared to the flat state, even when the bending radius was as small as 1 mm. We further demonstrated the application of ICCN dielectric in complementary circuits by fabricating flexible organic complementary inverters. Decent inverter performances were observed at low voltage (below 5 V). By presenting combined advantages of low surface roughness, high transparency, temperature resistance, flexibility, and solid-state ionic conductivity, ICCN thus possesses broad applications in future biodegradable, flexible, transparent, and all-solid low-voltage green electronics.

## Methods

### Materials

Sodium hypochlorite (NaClO) was purchased from Shanghai Titan Scientific Co., Ltd. TEMPO was purchased form Energy Chemical. (Tridecafluoro-1, 1, 2, 2-teterahydrooctyl) trichlorosilane (FOTS) was purchased from Sigma Aldrich. C8-BTBT was obtained from Suna Tech Inc. and PQT-12 from American Dye Source, Inc. NTCDI-F15 was synthesized following the literature procedure and purified by vacuum sublimation^61^.

### Fabrication of ionic conductive cellulose nanopaper (ICCN)

ICCN was prepared following the literature procedure^30^ with some modifications. TEMPO (100 mg) was ultra-sonicated in deionized water (95 mL) for at least 15 min in order to obtain uniform solution. Sodium bromide (NaBr, 659 mg) was dissolved in deionized water (65 mL), and then mixed with the uniform aqueous solution of TEMPO. Softwood pulp (6.5 g, dry weight) was suspended deionized water (80 mL) with violent agitation. Then, the mixed TEMPO/NaBr aqueous solution was added into the softwood pulp suspension followed by the addition aqueous solution of NaClO (38 mL, 12%). The pH of the mixed solution was monitored by using a pH meter (METTLER TOLEDO) and kept at 10.5 for 3 h by adding aqueous solution of sodium hydroxide (NaOH, 0.5 mol/L). Further, the product was purified by washing three times with deionized water. The washed product was diluted with deionized water and further dispersed into nanofiber using a blender machine. Then, the nanofibers were separated from the microfibers in the dispersion liquid by high speed centrifugation at 10,000 rpm for 30 min and the supernatant was collected. The supernatant was further tip-sonicated for 10 min in order to obtain the final uniform cellulose nanopaper pulp. The final cellulose nanopaper pulp was added into culture dish which has been pretreated with FOTS, and then dried naturally in semiconductor cleanroom. Finally, the solid-state ICCN was obtained and it could be easily peeled off from the culture dish.

### Fabrication of small molecule organic field effect transistors

For each OFET, a layer of ICCN was used as both dielectric and substrate simultaneously. One side of ICCN was spin coated with OTS toluene solution (7.5 μL/mL) at 5000 rpm for 60 s followed by heating at 60 ℃ for 1 h in a glovebox. C8-BTBT was thermal evaporated onto the OTS treated side of the ICCN at the rate of 0.1–0.3 Å/s. Au films (60 nm) were thermally evaporated through shadow masks as source-drain and gate electrodes, respectively, to form bottom gate, top-contact OFET devices. The channel length (*L*) and width (*W*) were 100 μm and 21 mm, respectively, unless specifically described. NTCDI-F15 OFETs devices were also fabricated by using the similar thermal evaporating method.

### Fabrication of polymer organic field effect transistors

PQT-12 chlorobenzene solutions (2.5 mg/mL) were spin cast on the top of a 40 μm thick OTS-modified ICCN for 2 times at the speed of 1500 r/min for 60 s and then annealed at 70 ℃ for 20 min to remove residual solvent. Au gate and source-drain electrodes were evaporated through shadow masks, respectively. The channel length (*L*) and width (*W*) were 100 μm and 21 mm, respectively.

### Fabrication of in plane gate organic field effect transistors

C8-BTBT were thermal evaporated on OTS treated ICCN (40 μm) through shadow mask. Au gate and source-drain electrodes were evaporated through shadow mask, respectively. The channel length (*L*) and width (*W*) were 100 μm and 21 mm, respectively.

### Fabrication of organic complementary inverters

NTCDI-F15 and C8-BTBT were thermal evaporated on OTS treated ICCN through shadow mask, respectively. Au gate (60 nm) and source-drain electrodes were also evaporated through shadow mask, respectively. Two gates of the n- and p-type OFETs were connected to form an input node while the source of the p-type OFET was connected to the drain of n-type OFETs to provide an output node.

### Characterization

The successfully introduction of sodium ions in ICCN was systematically investigated by FTIR spectroscopy (EQUINOX 55), EDS (Nova Nano SEM 450) and X-ray photoemission (XPS) (ESCALAB 250Xi). Thermal stability of ICCN was investigated by TG analysis. The surface morphologies of ICCN were investigated by atomic force microscope (AFM) (SEIKO SPA-300HV). The frequency dependent capacitances of ICCN was investigated by chemical impedance analyzer (IM3590). OFETs characteristics were tested in vacuum at room temperature using Keithley 4200 semiconductor system unless specifically described. The vacuum level for the testing of the devices is around 1 × 10^−4^ Pa.

### Data availability

All relevant data supporting the findings of this study are available from the corresponding author on request.

## Electronic supplementary material

Supplementary Information

## References

[1] Robinson BH (2009). E-waste: an assessment of global production and environmental impacts. Sci. Total Environ..

[2] Liu Q (2009). Chromosomal aberrations and DNA damage in human populations exposed to the processing of electronics waste. Environ. Sci. Pollut. Res. Int..

[3] Lei T (2017). Biocompatible and totally disintegrable semiconducting polymer for ultrathin and ultralightweight transient electronics. Proc. Natl Acad. Sci. USA.

[4] Zhu H (2016). Wood-derived materials for green electronics, biological devices, and energy applications. Chem. Rev..

[5] Irimia-Vladu M (2014). “Green” electronics: biodegradable and biocompatible materials and devices for sustainable future. Chem. Soc. Rev..

[6] Ionescu AM, Riel H (2011). Tunnel field-effect transistors as energy-efficient electronic switches. Nature.

[7] Wu X (2015). Thermally stable, biocompatible, and flexible organic field-effect transistors and their application in temperature sensing arrays for artificial skin. Adv. Funct. Mater..

[8] Zhang C, Chen P, Hu W (2015). Organic field-effect transistor-based gas sensors. Chem. Soc. Rev..

[9] Huang J (2013). Covalently functionalized double-walled carbon nanotubes combine high sensitivity and selectivity in the electrical detection of small molecules. J. Am. Chem. Soc..

[10] Huang J, Miragliotta J, Becknell A, Katz HE (2007). Hydroxy-terminated organic semiconductor-based field-effect transistors for phosphonate vapor detection. J. Am. Chem. Soc..

[11] Di CA, Zhang F, Zhu D (2013). Multi-functional integration of organic field-effect transistors (OFETs): advances and perspectives. Adv. Mater..

[12] Guo Y, Yu G, Liu Y (2010). Functional organic field-effect transistors. Adv. Mater..

[13] Gelinck GH (2004). Flexible active-matrix displays and shift registers based on solution-processed organic transistors. Nat. Mater..

[14] Sekitani T, Zschieschang U, Klauk H, Someya T (2010). Flexible organic transistors and circuits with extreme bending stability. Nat. Mater..

[15] Yi HT, Payne MM, Anthony JE, Podzorov V (2012). Ultra-flexible solution-processed organic field-effect transistors. Nat. Commun..

[16] Dumitru LM (2013). Plain poly(acrylic acid) gated organic field-effect transistors on a flexible substrate. ACS Appl. Mater. Interfaces.

[17] Tobjork D, Osterbacka R (2011). Paper electronics. Adv. Mater..

[18] Zhu H (2013). Biodegradable transparent substrates for flexible organic-light-emitting diodes. Energy Environ. Sci..

[19] Zhu H, Fang Z, Preston C, Li Y, Hu L (2014). Transparent paper: fabrications, properties, and device applications. Energy Environ. Sci..

[20] Zhong Q (2015). Paper-based active tactile sensor array. Adv. Mater..

[21] Olsson RT (2010). Making flexible magnetic aerogels and stiff magnetic nanopaper using cellulose nanofibrils as templates. Nat. Nanotechnol..

[22] Hu L (2013). Transparent and conductive paper from nanocellulose fibers. Energy Environ. Sci..

[23] Martins R (2011). Complementary metal oxide semiconductor technology with and on paper. Adv. Mater..

[24] Lim W (2010). Low-voltage indium gallium zinc oxide thin film transistors on paper substrates. Appl. Phys. Lett..

[25] Martins RF (2013). Recyclable, flexible, low-power oxide electronics. Adv. Mater..

[26] Zschieschang U, Klauk H (2015). Low-voltage organic transistors with steep subthreshold slope fabricated on commercially available paper. Org. Electron..

[27] Gaspar D (2014). Nanocrystalline cellulose applied simultaneously as the gate dielectric and the substrate in flexible field effect transistors. Nanotechonolgy.

[28] Bollström R (2009). A multilayer coated fiber-based substrate suitable for printed functionality. Org. Electron..

[29] Barhoum A, Samyn P, Ohlund T, Dufresne A (2017). Review of recent research on flexible multifunctional nanopapers. Nanoscale.

[30] Huang J (2013). Highly transparent and flexible nanopaper transistors. ACS Nano.

[31] Nogi M, Iwamoto S, Nakagaito AN, Yano H (2009). Optically transparent nanofiber paper. Adv. Mater..

[32] Fujisaki Y (2014). Transparent nanopaper-based flexible organic thin-film transistor array. Adv. Funct. Mater..

[33] Ortiz RP, Facchetti A, Marks TJ (2010). High-k organic, inorganic, and hybrid dielectrics for low-voltage organic field-effect transistors. Chem. Rev..

[34] Sun J, Zhang B, Katz HE (2011). Materials for printable, transparent, and low-voltage transistors. Adv. Funct. Mater..

[35] Kim SH (2006). Low-operating-voltage pentacene field-effect transistor with a high-dielectric-constant polymeric gate dielectric. Appl. Phys. Lett..

[36] Yoon MH, Facchetti A, Marks TJ (2005). Sigma-pi molecular dielectric multilayers for low-voltage organic thin-film transistors. Proc. Natl Acad. Sci. USA.

[37] Halik M (2004). Low-voltage organic transistors with an amorphous molecular gate dielectric. Nature.

[38] Robertson J (2004). High dielectric constant oxides. Eur. Phys. J. Appl. Phys..

[39] Pal BN, Dhar BM, See KC, Katz HE (2009). Solution-deposited sodium beta-alumina gate dielectrics for low-voltage and transparent field-effect transistors. Nat. Mater..

[40] Kim SH (2013). Electrolyte-gated transistors for organic and printed electronics. Adv. Mater..

[41] Dhoot AS (2006). Beyond the metal-insulator transition in polymer electrolyte gated polymer field-effect transistors. Proc. Natl. Acad. Sci. USA.

[42] Thiemann S (2014). Cellulose-based ionogels for paper electronics. Adv. Funct. Mater..

[43] Cunha I (2017). Reusable cellulose-based hydrogel sticker film applied as gate dielectric in paper electrolyte-gated transistors. Adv. Funct. Mater..

[44] Cho JH (2008). Printable ion-gel gate dielectrics for low-voltage polymer thin-film transistors on plastic. Nat. Mater..

[45] Yuan H (2009). High-density carrier accumulation in ZnO field-effect transistors gated by electric double layers of ionic liquids. Adv. Funct. Mater..

[46] Wang CY (2015). Stable low-voltage operation top-gate organic field-effect transistors on cellulose nanocrystal substrates. ACS Appl. Mater. Interfaces.

[47] Jung YH (2015). High-performance green flexible electronics based on biodegradable cellulose nanofibril paper. Nat. Commun..

[48] Zschieschang U (2011). Organic electronics on banknotes. Adv. Mater..

[49] Li Y (2012). Solution-processed organic crystals for field-effect transistor arrays with smooth semiconductor/dielectric interface on paper substrates. Org. Electron..

[50] Lizundia E, Vilas JL, Leon LM (2015). Crystallization, structural relaxation and thermal degradation in Poly(L-lactide)/cellulose nanocrystal renewable nanocomposites. Carbohydr. Polym..

[51] Colom X, Carrillo F (2002). Crystallinity changes in lyocell and viscose-type fibresy caustic treatment. Eur. Polym. J..

[52] Lizundia E (2016). Cu-coated cellulose nanopaper for green and low-cost electronics. Cellulose.

[53] Nti F, Han JI (2017). Layered Na_2/3_ Ni_1/3_ Mn_2/3_ O_2_ as electrode material with two redox active transition metals for high performance supercapacitor. J. Alloy Compd..

[54] Rhee SW, Yun DJ (2008). Metal–semiconductor contact in organic thin film transistors. J. Mater. Chem..

[55] Yadav S, Kumar P, Ghosh S (2012). Optimization of surface morphology to reduce the effect of grain boundaries and contact resistance in small molecule based thin film transistors. Appl. Phys. Lett..

[56] Herlogsson L, Crispin X, Tierney S, Berggren M (2011). Polyelectrolyte-gated organic complementary circuits operating at low power and voltage. Adv. Mater..

[57] Yokota T (2016). Ultraflexible organic photonic skin. Sci. Adv..

[58] Sadek A, Ismail K, Armstrong MA, Antoniadis DA (1996). Design of Si/SiGe heterojunction complementary metal-oxide-semiconductor transistors. IEEE Trans. Electron Devices.

[59] Isogai A (2013). Wood nanocelluloses: fundamentals and applications as new bio-based nanomaterials. J. Wood Sci..

[60] Moon RJ, Martini A, Nairn J, Simonsen J, Youngblood J (2011). Cellulose nanomaterials review: structure, properties and nanocomposites. Chem. Soc. Rev..

[61] Katz HE (2000). A soluble and air-stable organic semiconductor with high electron mobility. Nature.

