# Supplementary material for: Intrinsically ionic conductive cellulose nanopapers applied as all solid dielectrics for low voltage organic transistors
Source: Nat Commun. 2018 Jul 16;9:2737. doi: 10.1038/s41467-018-05155-y (PMC6048164; doi:10.1038/s41467-018-05155-y)
Supplement: Supplementary file 1 — Supplementary Information [file 41467_2018_5155_MOESM1_ESM.pdf]

Supplementary Information for

Intrinsically ionic conductive cellulose nanopapers applied as all solid dielectrics for low voltage organic transistors

Dai et al.

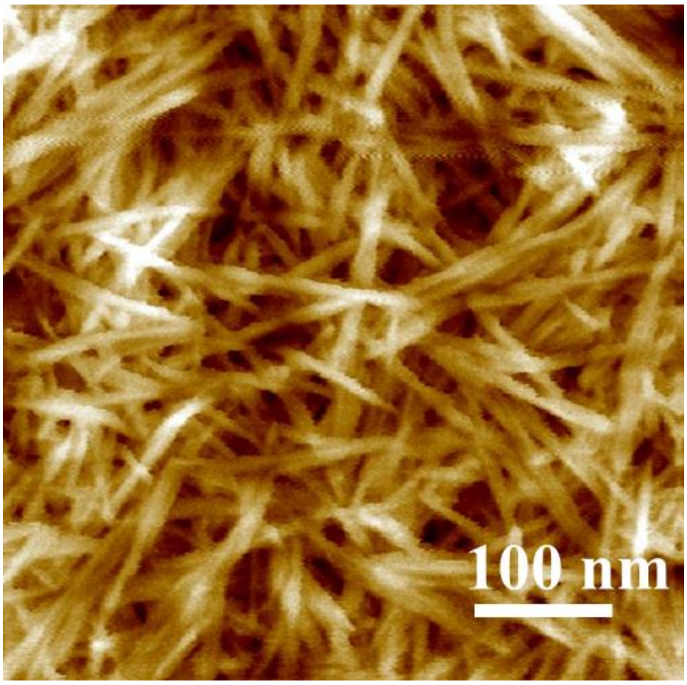

Supplementary Figure 1 | AFM measurement. AFM image of a diluted nanocellulose solution on silicon substrate.

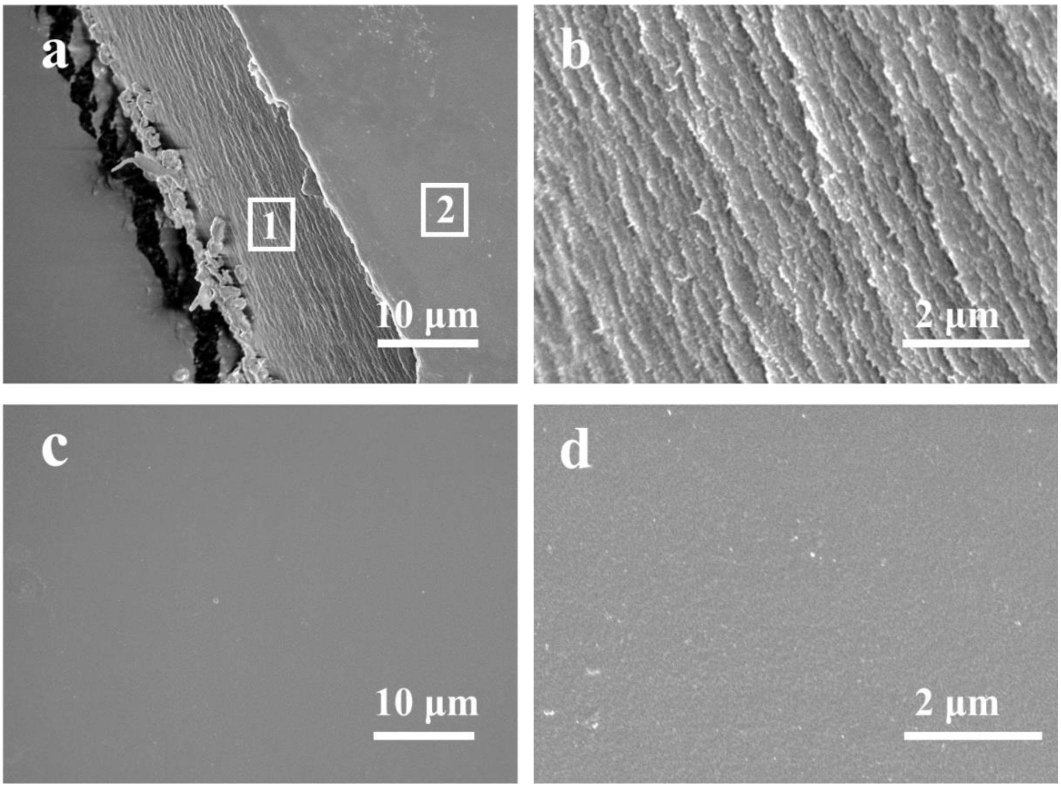

Supplementary Figure 2 | SEM characterization. (a) SEM image of ICCNs. (b) Enlarged image of region “1” in (a). (c) and (d) enlarged images of region “2” in (a).

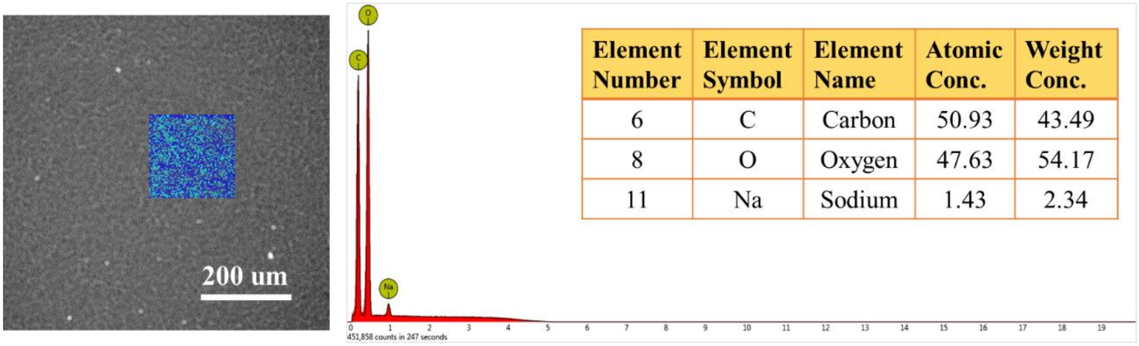

Element Number	Element Symbol	Element Name	Atomic Conc.	Weight Conc.
6	C	Carbon	50.93	43.49
8	O	Oxygen	47.63	54.17
11	Na	Sodium	1.43	2.34

Supplementary Figure 3 | EDS characterization. EDS results of a 40 μm-thick ICCN.

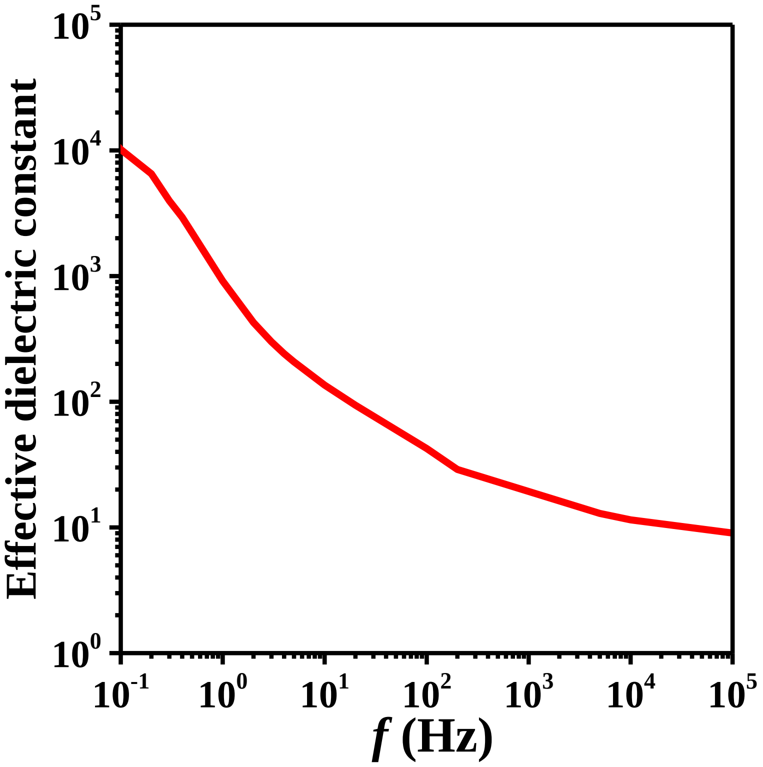

Supplementary Figure 4 | Frequency-dependent effective dielectric constant. Frequency-dependent effective dielectric constant of a 40 μm-thick ICCN measured in a metal/insulator/metal (Au/ICCN/Au) structure.

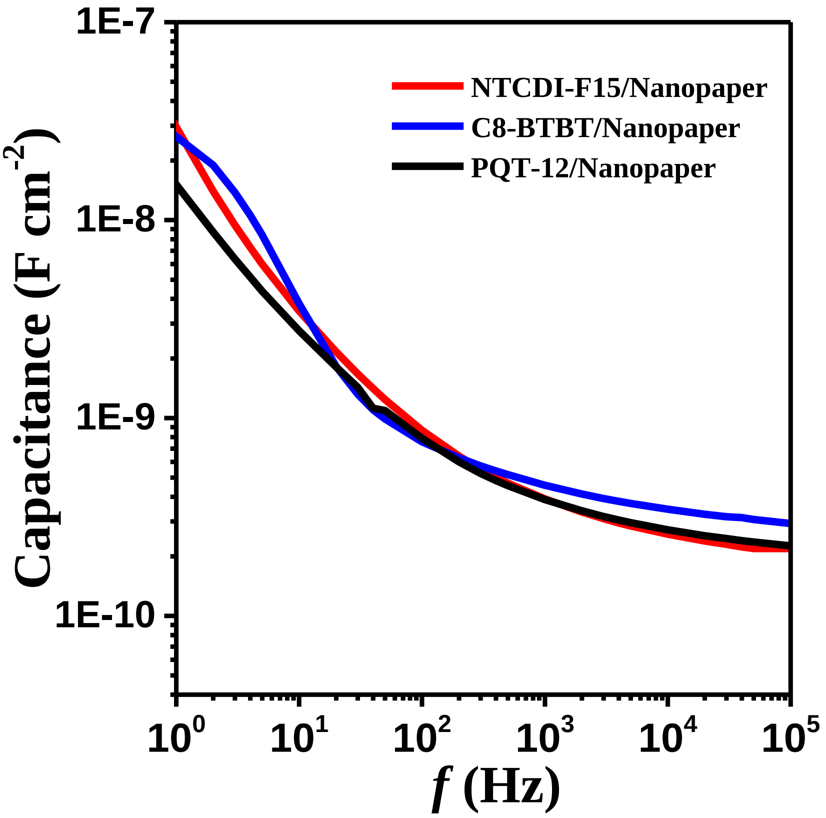

Supplementary Figure 5 | Frequency-dependent effective capacitances of ICCNs. Frequency-dependent effective capacitance (C-*f*) of 40 μm-thick ICCNs measured in a metal/insulator/semiconductor/metal structure.

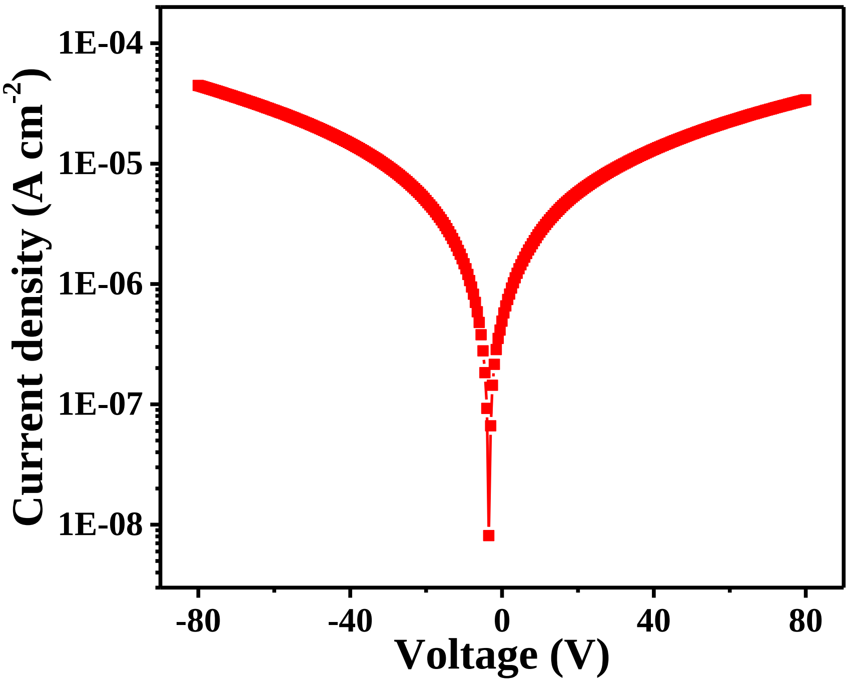

Supplementary Figure 6 | Voltage dependence current density of ICCNs. Voltage dependence current density of 40 μm-thick ICCNs in metal/insulator/metal (Au/ICCN/Au) structure.

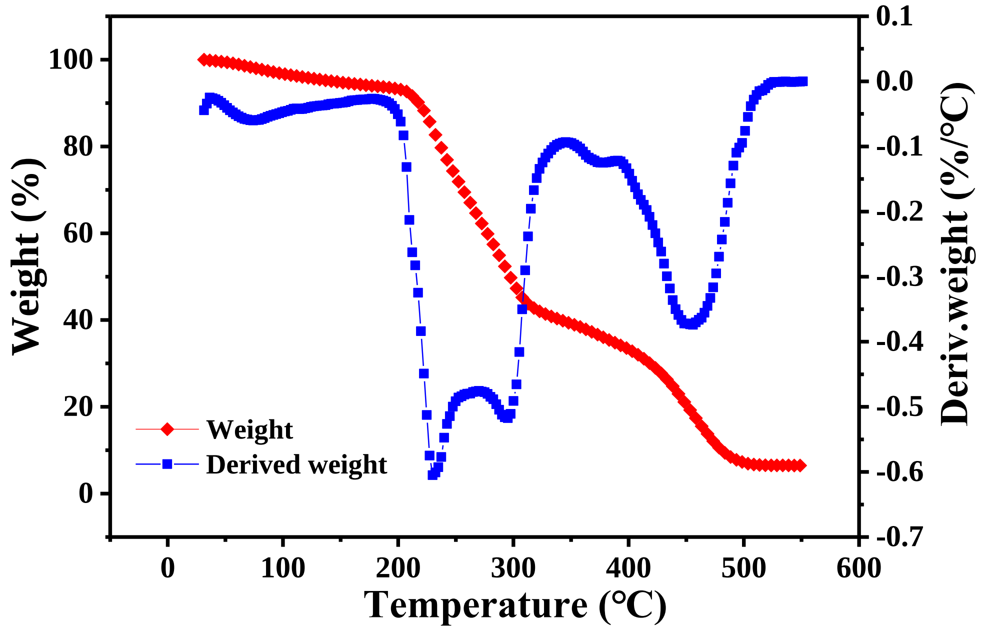

Supplementary Figure 7 | TG analysis of ICCN. The thermogravimetry spectrum for a 40 μm-thick ICCN. The sample was heated from 25 to 550°C at a rate of 5 °C min^{-1}.

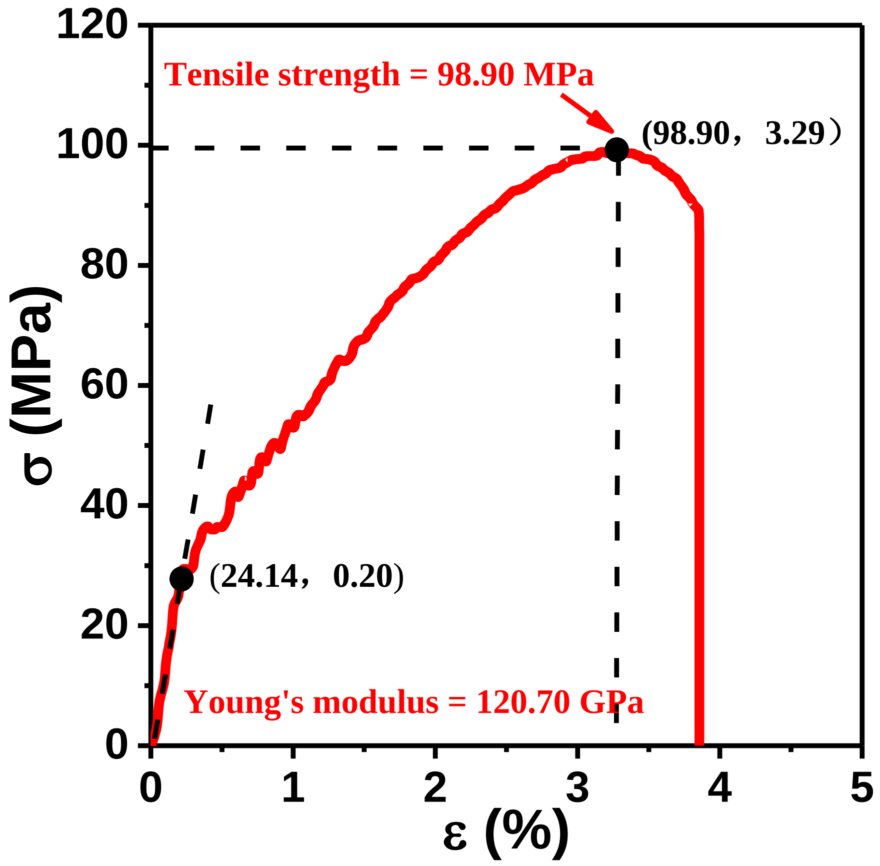

Supplementary Figure 8 | Mechanical property of ICCNs. Typical stress-strain (σ-ε) curve of 40 μm-thick ICCNs. Young's modulus of ICCNs was calculated to be 120.70 GPa, and the tensile strength was 98.90 MPa.

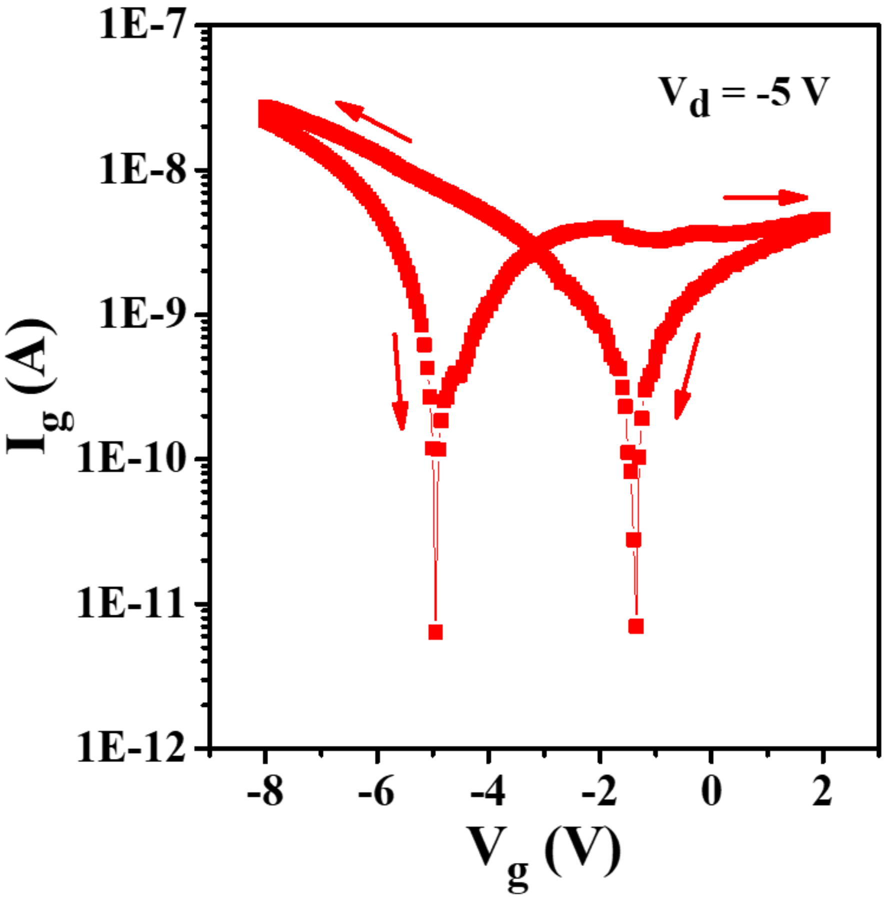

Supplementary Figure 9 | Leakage current of ICCN based OFETs. Gate leakage current of C8-BTBT OFETs with 40 μm-thick ICCNs acting as dielectrics.

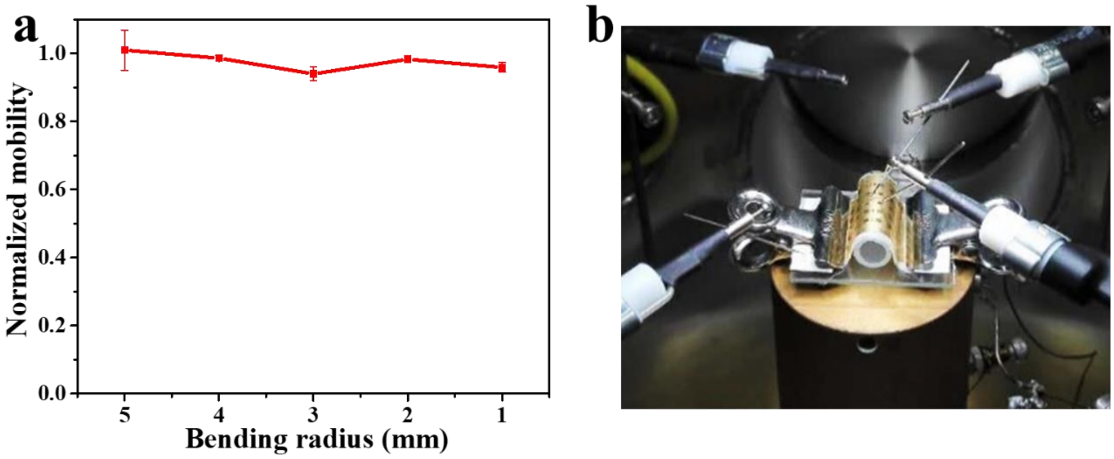

Supplementary Figure 10 | Bending tests on flexible OFETs. (a) Normalized mobility (normalized to the initial mobility measured in the flat state) as a function of the bending radius. Mobility values were extracted from the saturation region. Error bars represent standard errors from 5 times independent tests of a device. (b) The picture depicts implementation of this bending test.

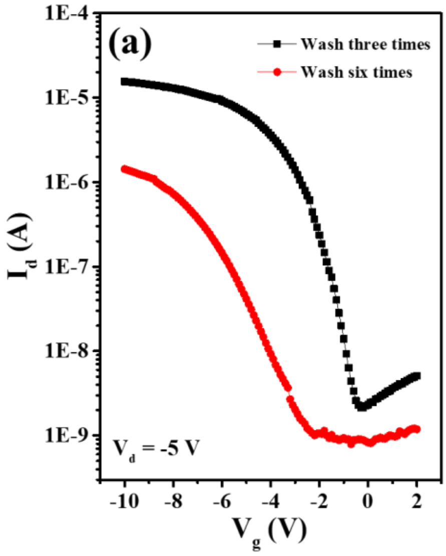

(b)

Element Number	Element Symbol	Element Name	Atomic Conc.	Weight Conc.
6	C	Carbon	50.93	43.49
8	O	Oxygen	47.63	54.17
11	Na	Sodium	1.43	2.34

(c)

Element Number	Element Symbol	Element Name	Atomic Conc.	Weight Conc.
6	C	Carbon	51.55	44.10
8	O	Oxygen	47.11	53.69
11	Na	Sodium	1.35	2.20

Supplementary Figure 11 | Tansfer curves of ICCN-based C8-BTBT OFETs. (a) Transfer curves of C8-BTBT OFETs based on 40 μm-thick ICCNs. ICCNs were prepared from nanocellulose solutions with different washing times. EDS results of 40 μm-thick ICCNs prepared from three-time-washed nanocellulose solution (b) and six-time-washed nanocellulose solution (c), respectively.

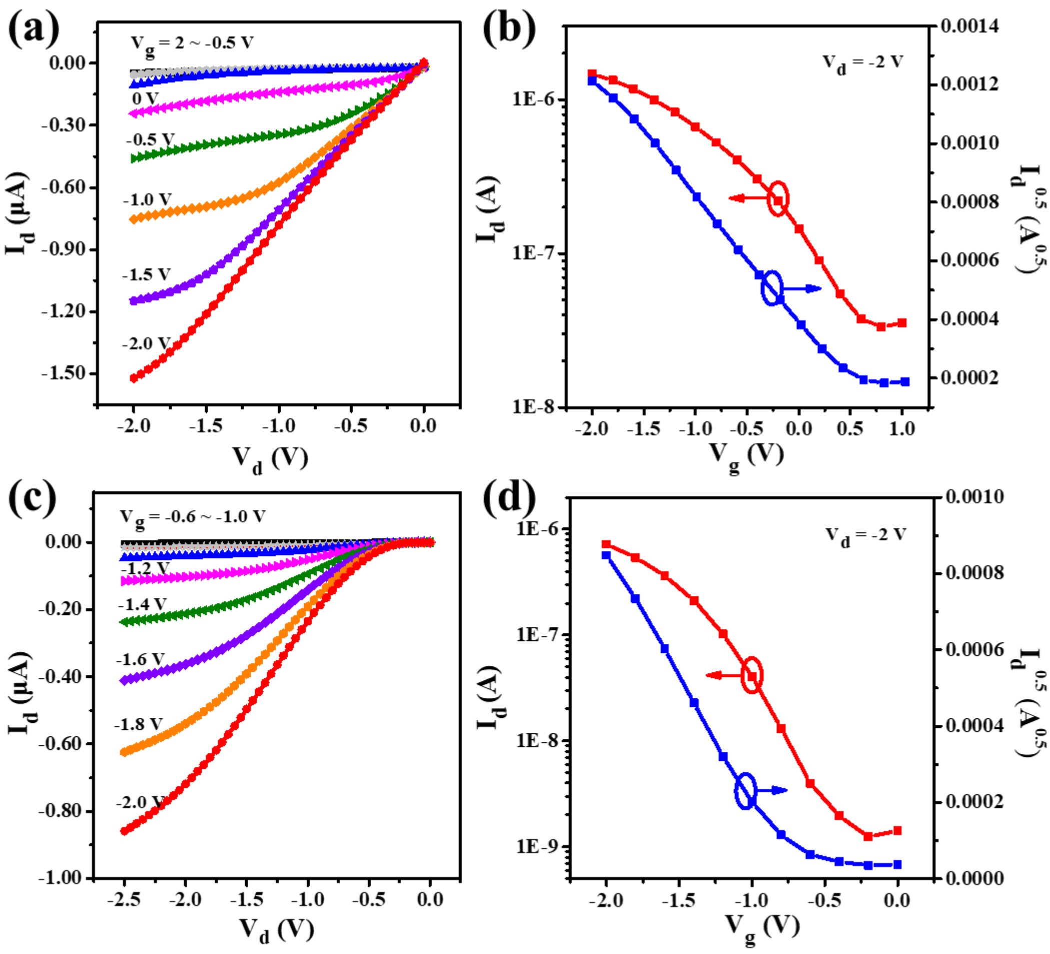

Supplementary Figure 12 | OFETs performances. (a) Output I_d–V_d curves and (b) transfer characteristics curves (I_d–V_g) of a CYTOP encapsulated C8-BTBT OFET with 80 μm-thick ICCN acting as dielectric. (c) Output I_d–V_d curves and (d) transfer characteristics curves (I_d–V_g) obtained from the same device in vacuum before CYTOP encapsulation.

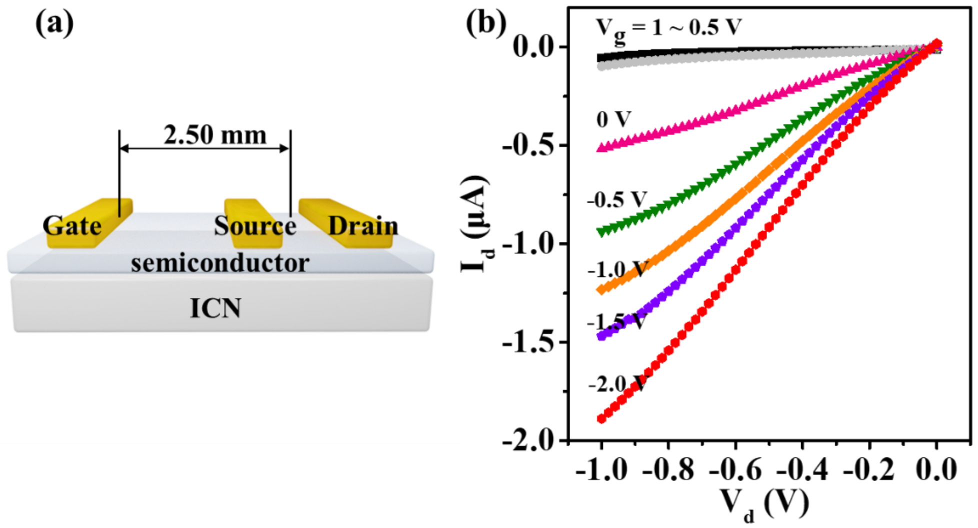

Supplementary Figure 13 | In-plane gate OFETs performance. (a) Schematic illustration and (b) output I_d-V_d curves of in-plane gate C8-BTBT OFETs using ICCNs acting as dielectrics. These in-pane gate devices were tested in air.

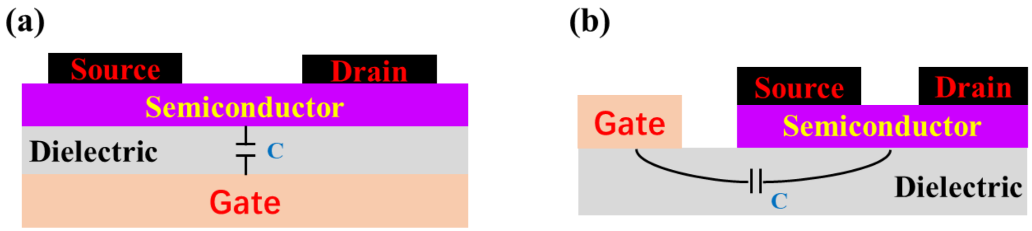

Supplementary Figure 14 | Schematics of different capacitive coupling effect. (a) Vertical capacitive coupling effect. (b) Lateral capacitive coupling effect.

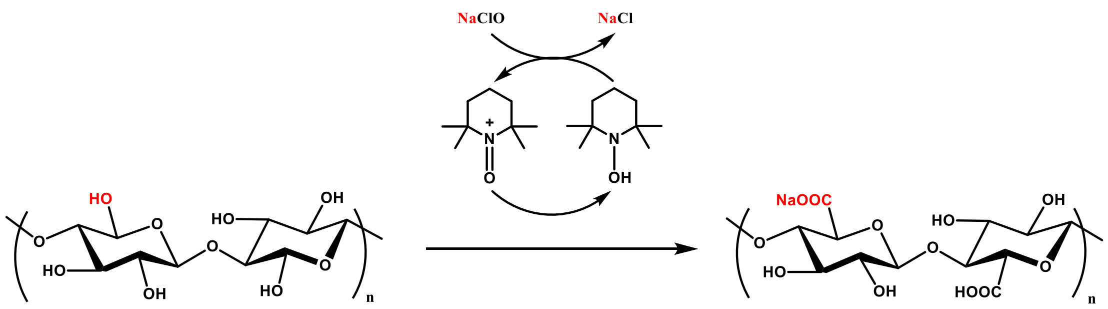

Supplementary Figure 15 | Chemical reaction process. Preparation of nanocellulose by TEMPO oxidation process.

Supplemenatry Tables

Supplementary Table 1. Key parameters of the organic complementary inverter.

V_{DD} [V]	Gain [V/V]	NM_H[a)] [V]	NM_L[b)] [V]	TW[c)] [V]	V_l[d)] [V]
2	2.8	0.90	0.16	0.50	1.24
3	4.1	1.16	0.10	0.78	1.85
4	5.4	1.62	0.03	0.90	2.49
5	7.6	1.75	0.32	0.95	3.15

[a)]$NM_H=V_{OH}-V_{IH}$, high noise margin; [b)]$NM_L=V_{IL}-V_{OL}$, low noise margin; [c)]$TW=V_{IH}-V_{IL}$, transition width; d)$V_l=V_{OH}-V_{OL}$, logic swing. Here, V_{OH}, V_{IH}, V_{IL} and V_{OL} were assessed with VTC curves shown in Figure 7b.
